# Inference to the best action and its basis in clinical expertise

**DOI:** 10.3389/fpsyg.2023.1032453

**Published:** 2023-11-27

**Authors:** Mark Fedyk, Jessica Draughon Moret, Nicolas T. Sawyer

**Affiliations:** ^1^Division of General Internal Medicine and Bioethics, Department of Internal Medicine, School of Medicine, University of California, Davis, Davis, CA, United States; ^2^Betty Irene Moore School of Nursing, University of California, Davis, Sacramento, CA, United States; ^3^Department of Emergency Medicine, School of Medicine, University of California, Davis, Sacramento, CA, United States

**Keywords:** inference to the best explanation, clinical expertise, nursing science, medical expertise, scientific models, clinical models, philosophy of practice

## Abstract

Can contemporary cognitive science explain clinical expertise? We argue that the answer could be “no.” In support of this, we provide an analysis of two of the most essential expressions of clinical expertise in nursing and medicine, the ability to run a code blue and the ability to diagnose congestive heart failure. We show how it makes sense to treat both as examples of what we call inference to the best action, and we then argue that two of the standard explanatory paradigms of cognitive science — the Humean and Bayesian paradigms — are unable to provide a plausible analysis of inference to the best action.

## 1 Introduction

Can contemporary cognitive science explain clinical expertise? We argue that the answer could be “no.”

To accomplish this, the paper is split into two parts, the constructive first two-thirds of the paper and then a critical final third. In the constructive sections, we provide an analysis of two of the most essential expressions of clinical expertise in nursing and medicine, the ability to run a code blue and the ability to diagnose congestive heart failure. Since our aim is to provide a more or less unified analysis of each, we show how it makes sense to treat both as examples of what we call *inference to the best action*. Or, put the other way around, we suggest that the ability to make inferences to the best action in clinical contexts *is* what clinical expertise is for — and that running a code blue and diagnosing congestive heart failure are both paradigmatic cases of inferences to the best action.

The concept of inferences to the best action is logically tied to a theory of the cognitive basis of these judgments: we contend that it is knowledge of (different kinds of) *models* that is the source of inferences to the best action. It is here that we locate the tension between some of the organizing principles of contemporary cognitive science and the analysis that we develop in the constructive sections of the paper. In the critical section of the paper, we begin by offering a schematic representation of popular Humean and Bayesian principles about cognitive architecture. This schematic representation allows us to precisely locate the points where our analysis of inference to the best action contradicts certain principles.

If our analysis of clinical expertise as knowledge of the kinds of models which yield inferences to the best action has meaningful abductive — in the Peircean sense of the term^1^ — weight, there is some tension between standard, and deep, assumptions in contemporary cognitive science and efforts to develop a cognitive science of clinical expertise. Therefore it should not be taken for granted that mainstream cognitive science contains the resources to explain clinical expertise.

Our argument ought not appear particularly surprising given the history of psychology. Peirce stressed the generative, constructive nature of abductive thinking. He explains abduction as “reasoning from surprise to [knowing how to conduct scientific] inquiry” (Peirce, 2021, pp. 907–908) — and in our analysis of the cognitive expertise of skilled clinicians, we stress a similar connection between expertise-driven thinking and skilled, useful clinical action. This approach to abduction is a contrast with the more recent trend — seemingly initiated by Gilbert Harman (Harman, 1965) — of using “abductive” and “abduction” to denote only efforts to come up with a true explanation, and for that, an explanation that takes the form of a linguistic description of a set of pre-existing facts or observations. As will soon become clear, we think there is good reason for cognitive scientists to stick with the older, Piercean meaning, according to which abduction is in some important sense, “thinking for doing.”

Finally, a word about context and our choice of stylized examples. We have chosen our examples not because we think they are paradigmatic about nursing and medicine; we are not trying to offer an analysis of either medical expertise or nursing expertise as general categories of specialized knowledge.^2^ Rather, our examples are chosen because they illustrate the materiality of *enacting* interprofessionalism in the clinical setting, the materiality of *making* a diagnosis in a clinical setting, and the ways in which cognition must adapt to both. Puting the same clarification another way, our examples are chosen to try to illustrate something important about the material situatedness of clinical expertise (Haraway, 1988), and the challenges that such analyses create for cognitive science. The practice in cognitive science of studying the parts and processes of the cognitive system in abstraction from the complex ways in which environments create affordances for both expert inference and action may — following, for instance (Rogoff, 1990) — entail a non-trivial scientific cost. We suggest that an effective way of bringing this cost into relief is to closely examine clinical expertise as it is put into practice.

## 2 Cardiac arrest and resuscitation outside of the ED

Let us start with perhaps the most archetypal emergencies seen in a hospital: a code blue. A code blue is called when a patient, already admitted to a hospital ward, experiences respiratory or cardiac arrest. A team of nurses, physicians, and technicians will surround the patient, and work together to try to achieve return to spontaneous circulation (ROSC), which is when the patient can breathe on their own and has a stable heart beat unaided by any medical devices. Because of the intensity of the action involved in running one, code blues are a frequent trope of TV medical dramas.

The arrest is often anticipated. Patients near the point of respiratory or cardiac arrest usually show both signs and symptoms of clinical deterioration. Signs include such information as a cardiac monitor showing acute hypoxia, hypercarbia, an unstable tachyarrhythmia or bradyarrhythmia. On physical exam, patients may exhibit diaphoresis, acute shortness of breath, agitation, profound somnolence, or unconsciousness. Symptoms include what the patient can report about themselves, and thus may or may not be present, depending on the patient’s level of consciousness or the patient’s ability to communicate. But the code blue does not start until the patient stops breathing or their heart stops.

Technically, a code blue is a repertoire of interdependent perceptual abilities and actions that are engaged or enacted in different combinations and sequences depending upon the outcome of previous actions, changes in the patient’s status, or any number of otherwise unforeseeable occurrences that can happen during a code blue. Because of how dynamic code blues can be, to learn what must be done during a code blue, nurses and physicians use simulation training.

Importantly, a code blue cannot be performed outside of a hospital. Code blues are materially linked to the situation in which they are enacted by way of the *appropriate* use of the advanced medical equipment required to execute the relevant actions: you need a cardiac monitor, a crash cart which contains two large bore IV lines, IV fluids, advanced cardiac life support (ACLS) medications, advanced airway equipment, a defibrillator, and the ability to keep the patient’s body prone and firmly supported. More importantly, you have a team of trained professionals in the room who understand their roles and responsibilities well enough for them to work together as a cooperative unit, capable of changing actions in concert very quickly and often without explicit direction. This knowledge is thus causally efficacious, in a fast-paced and rapidly changing context.

This team which enacts a code blue includes a physician whose function is to lead the other participants, and in so doing consider the probable causes of the respiratory or cardiac arrest, order the appropriate resuscitation medications to be administered at the appropriate times and at appropriate dosage levels, perform procedures such as intubation and placing central venous line, analyze STAT laboratory studies and the electrical activity of the heart as seen on the cardiac monitor, as well as assess and reassess the patient on an ongoing basis. But the sequence of actions that actually occurs during a code blue is usually a by-product of distributed decision-making, as responsibility for either directing or taking the next action in a code blue can fall to whoever has the most important information at that moment in time.

Nurses will perform CPR, place IV and central lines, assist with family members, who can be an important source of medical history, and otherwise ensure that the team maintains a degree of coordination. This is important because a full code blue team can include respiratory therapists, licensed clinical social workers, medical scribes, and health sciences students from various specialties and at varying levels of education. The team itself can be formally organized as a hospital’s in-house resuscitation team, or it may be formed *ad hoc* if the situation requires it.

The point of these details is to make it clear that code blues are deeply *situated* — meaning, code blues can only occur in situations that are specifically prepared to facilitate the enactment of the code blue, including both the technology and the trained personnel. The enactment of a code blue and its situational dependencies are tightly coupled together: the former is only possible in the presence of the latter.

Here is a different way to make the same point. Continuous chest compressions are always necessary following cardiac arrest, and the chest must be compressed to a depth greater than 38 mm to maintain constant cardiac output and cerebral blood flow, which maximizes the chances of the patient recovering without significant disability should they survive cardiac arrest. Since compressing a chest to that depth requires a lot of force, an experienced nurse will ensure that a small footstool is placed close to the crash cart, or is otherwise easily accessible to the people performing CPR, so that it can be used by whichever team member is delivering the chest compressions. Standing on this stool reduces the moment of inertia required for every chest compression, reducing fatigue, prolonging the team’s ability to provide effective chest compressions. But even with the stool, delivering chest compressions is quickly exhausting work, and a code blue can last 20 min or more. Members of the code blue team must therefore train to be able to fluidly rotate through a small roster of individuals who are either delivering chest compressions or resting from the exertion, preparing to rotate back in.

Of course, the point here is not that chest compressions are impossible to perform outside of the context of a code blue. Nor that it is impossible to monitor and record an ECG absent of the conditions leading to the onset of a code blue. People can obviously be resuscitated from cardiac or respiratory arrest in many ways and in many different situations. The point, rather, is that what makes a code blue the sequence of actions that it is comes from bundling together a collection of independently medically useful techniques, measures, abilities, and skills together into something that — not just aims to, but at least sometimes does in fact — achieve a particular end, namely achieving return of spontaneous circulation. Even more importantly, this end can be achieved despite the code blue being enacted in a dynamic, constantly changing reality, and where the application is itself a highly coordinated social activity.

### 2.1 Clinical expertise as knowledge of PIO models

Because of this, we want to suggest that when nurses and physicians learn what they must do when responding to a code blue, what they are learning is a *process of interventions and outcomes* (“PIO”) model: what they come to know is more complicated than a set of scripts or heuristics or checklists, because the relevant knowledge supports complex counterfactual inferences, and where — crucially — many of the essential the conditions upon which the utility of the counterfactual inferences depend cannot be known in advance and therefore cannot be expressed as branch on some predefined decision tree. The need to discover what the situation is and why “working through it” is also a reason why this expert knowledge is unlikely to take the form of a general theory of, e.g., the domain of cardiac function. We suggest that it is best characterized as knowledge of a model — or, more specifically, a particular species of model — again, a model of a process of interventions and outcomes.

Models are well-studied in philosophy of science. They are usually treated as abstract representations that function as useful approximations, idealizations, or abstractions for complex natural phenomena (Cartwright et al., 2001; Ankeny et al., 2011; Nersessian, 2012; Weisberg, 2012). Constructing and using a model provides a tractable way of thinking about something that escapes direct descriptions or interaction.

That is the same justification we use for introducing the concept of *PIO models*: learning PIO models gives people knowledge of and access to complex, interacting, multi-level and often interdependent processes.^3^ Resuscitation of a patient following cardiac arrest by way of code blue requires establishing and maintaining beneficial alignment between multiple different social processes and an even larger number of physiological processes.

At this point it is natural to ask, what is a code blue a model *of*? A bit of semantic housekeeping allows us to address this question. When code blues are enacted — when a resuscitation team is working to achieve ROSC somewhere in a hospital — they are enacting the model. The model’s object — that is: what the model is a model *of* — are processes and interventions that can generate an idealized outcome: after not too much effort, the patient’s heart beats on its own, and the patient regains consciousness without any serious, long-term impairments. Or, in the general case, PIO models *model* processes that should cause the ideal outcome where a patient is successfully treated.

Returning to ideas raised two paragraphs before, the reason it makes sense to call an enactment of a code blue an enactment of a PIO model is that, as the efforts to restore spontaneous circulation unfold, new information and events are constantly emerging and occurring, and the participants in the model must react intelligently, flexibly, and without much deliberate effort to continually coordinate and orient their actions. Much of this is counterfactual — indeed, causal — thinking, that flows into any number of clinically efficacious interventions. For instance, it may include an understanding that, if the person performing chest compression tires too quickly, they may be knocked out of the way by someone who is able to step in and deliver sufficiently deep compressions. Or, put more generally, all members of a code blue team will be trained to be collectively sensitive to any number of counterfactual scenarios, and have shared understanding about what actions to take — either individually or in concert — should any of these scenarios occur.

Even if someone learned everything about all the clinical processes and medical interventions associated with code blues that can be learned, responding to a patient in cardiac arrest by deductively trying to figure out what to do in a stepwise fashion, guided by an articulable set of propositions that themselves are logically interconnected, would be too slow for this expertise to have any clinical utility. But at the same time, a code blue is too complex for people to enact the code blue by memorizing decision trees, if-then scripts, or heuristics. Hence, the suggestion that when a code blue is enacted, clinicians are enacting a PIO model.

Finally, a clarification. We do not mean to reintroduce the distinction between system-1 and system-2 cognitive processes (Stanovich and West, 2000; Morewedge and Kahneman, 2010; Evans and Stanovich, 2013). Rather, we want to suggest that the use of models to navigate code blue situations is an example of fast, effective, but not automatic reasoning — and where, importantly, both the speed and the efficacy of the reasoning is explained to a meaningful degree by the tight coupling of the “conceptual objects” that clinicians are relying upon and the material configuration of the space. This is what allows us to posit that skilled clinicians are not relying on decision trees, if-then scripts, and heuristics: these various “conceptual objects” are (partially; they may be reconfigured in situ) configured in advance, and for that reason they would appear fragile or insufficient in the face of the novelty and complexity of most real-world code blue scenarios. The deeper reason for this is that decision trees, if-then scripts, and heuristics relate to reality through correspondence, where what is needed is *coordination* (not just) between the relevant conceptual objects and complex causal reality; this is a point that we will return to in more detail below.

### 2.2 Congestive heart failure and diagnostic models

These are still fuzzy observations. To further refine them, we’d like to introduce another kind of model, namely, a diagnostic model. *Diagnostic models are patient-specific models of the patient’s actual, too complex to observe or describe, physiology*. Whereas PIO models are models of clinical processes and interventions that can generate ideal outcomes, diagnostic models are models of otherwise cognitively inaccessible physiological processes that are occurring in just one patient. Diagnostic models are more flexible than PIO models: their content evolves in concert with a patient’s pathophysiology; often the information on a patient’s chart is a shorthand for the most important elements of the most recent diagnostic model for that patient.

Congestive heart failure (CHF) is a good illustration of this new concept. CHF is a complex clinical syndrome that results from either functional or structural impairment of ventricles resulting in symptomatic left ventricle dysfunction. It is one of the most common diagnoses in the United States for patients over the age of 65, and it is one of the leading causes of death worldwide. Crucially, CHF does not have a single cause (e.g., longstanding uncontrolled hypertension) and it can produce various secondary effects (e.g., impaired kidney function resulting in lower extremity edema) that must be managed in the course of treating CHF. There is also no single test that can be used to diagnose CHF, and there is no cure — at least not in the sense of taking a pill that leads to the return of normal cardiac function regardless of underlying etiology.

Understanding how CHF is diagnosed and subsequently managed is made a bit easier if we introduce the concepts of homeostasis and allostasis. Homeostasis refers to the normal operating range of the body’s various physiological systems, and allostasis the capacity of both these and other systems to return the first set of systems to normal operating range under conditions of stress: allostatic resources function to induce a return to homeostasis when the body’s homeostatic operations are disrupted or stressed. CHF is a by-product of insufficient allostatic resources: the body is losing its ability to compensate for an increasingly weakening heart, generating decompensations in many other physiological systems.

CHF is consequently diagnosed incrementally. Certain physical exam findings — such as peripheral pitting edema — may be strongly dispositive. But the diagnosis is built on the basis of collecting a comprehensive set of evidence, which can include measures of the heart’s ejection fraction (the volume of blood pumped out of a ventricle divided by the volume of blood in the ventricle at the end of diastolic filling), imaging showing cardiomegaly (an enlarged heart), taking a detailed patient and family history, and various other indicators.

Indeed, a preliminary model of the patient’s allostatic capacities has to be generated before CHF can be meaningfully entertained as a diagnosis. This is why it is in fact a deep mistake to conceptualize the process of differential reasoning that physicians engage in when making a diagnosis as *only* a process of elimination: it is also a process by which a diagnostic model for a particular patient gets constructed and then subsequently updated. And the point of introducing the concept of a diagnostic model is to account for the fact that rarely if ever do patients present with illnesses that correspond to textbook definitions of the same. In order to treat a patient, a cognitive object must be created that can be shared by members of the clinical team, and which is specific enough to the patient to be a useful guide to the patient’s care. Thus, a diagnostic model of a patient’s CHF will represent the specific way in which the patient’s heart is failing, the various systematic effects of cardiac failure, and any number of further comorbidities or complications. Furthermore, since illness is never static, any useful diagnostic model for a patient must be constantly reconciled with the patient’s physiology as the patient improves, deteriorates, or otherwise changes.

A common trope among physicians is that diagnosis is detective work: when a sick patient presents to a hospital, it is obvious that something has gone wrong. But the detective metaphor is misleading in one important respect. Detectives tell you who committed the murder; providing a model of how the crime unfolded is usually left to the forensics team. A useful diagnosis combines both of these elements: it indicates the cause of illness, but it also provides a patient-specific insight of the their own physiology: *their* heart is failing in this particular way, say, for example because they had a large acute myocardial infarction leading to decreased contractility in the affected heart muscle, leading to a reduced ejection fraction, lung edema and thus the patient’s complaint of shortness of breath.

Further details. A diagnostic model aims not to just provide a useful abstraction and simplification of the otherwise too complex to grasp physiology of the patient. Diagnostic models also mediate between the patient and a large number of additional physiological, institutional, or social factors. While, generally speaking, a patient with heart failure needs furosemide, the specific care for each patient is individualized and must take into account the severity of their disease, their comorbidities, their belief systems, the availability of equipment and personnel, availability of consultants depending on time of day, affability between coworkers as well as consultants, the ability of the patient to participate in their own care, and a very large number of additional factors. The function of a diagnostic model is to establish patterns of coordination action, taking all of these factors into account. Physicians therefore construct diagnostic models that are fit — and thus can be used within — the clinical context in which care is being provided. Like PIO models, diagnostic models are deeply situated, thus.

Another example may further clarify the concept of a diagnostic model. Patients with Type 1 diabetes do not secrete sufficient insulin from their pancreas. As insulin is the hormonal “gatekeeper” that facilitates the entry of glucose from the bloodstream into cells, in the absence of insulin, blood glucose levels can become notably elevated. The absence of glucose in the patient’s cells leads to the intracellular formation of an alternative source of energy, namely ketone bodies. While ketones can be used to sustain metabolism, they also cause the blood to become acidic which, if left untreated past a certain pH, leads to the state known as diabetic ketoacidosis (DKA). There are some patients with Type1 diabetes who have never been in DKA while others present to the emergency department in DKA almost weekly. Why? There are multiple reasons a patient can be tipped into a state of DKA even if they are using their insulin as prescribed. Examples include infection, a stroke or heart attack, intoxication with alcohol or stimulant use, and pregnancy. Other factors are social. Insulin needs to be continuously refrigerated which can be impossible for an unhoused patient. Patients with severe mental health disorders such as schizophrenia are usually too disorganized to manage their own insulin regimen which requires multiple daily doses, correction of blood sugar for meals and a different, longer acting insulin to be administered once before bedtime. Because of all of these differences, a unique diagnostic model will be constructed for each and every patient who presents at the emergency department with DKA — a diagnostic model useful for getting one patient out of DKA will be inapplicable to any other patient.

### 2.3 Clinical expertise and federations of PIO models

Both PIO models and diagnostic models are deeply situated. But there is an important difference between these two kinds of models: PIO models are *governed* in a way that diagnostic models are not — and cannot be. Diagnostic models are freely constructed by clinicians with the aim of generating a usable simplification of a specific patient’s pathophysiology, one that allows for effective action in the face of often inscrutable physiological complexity. PIO models, by contrast, are standardized, as they provide the epistemic foundations for a very large number of coordinated clinical behavior. The easiest way to see this is to understand that the team of clinicians who enacts a code blue may have never worked together before.

Nurses and physicians need to know many more PIO models than just the one which is useful for guiding the enactment of a code blue. Yet, there is no meta-model or meta-theory that unifies all the PIO models that must be grasped by members of a clinical team in order for them to be effective. Where does the unity of this knowledge come from? And how is this knowledge standardized?

We suggest that it is helpful to think of the relevant collection of PIO models as a federal entity. Different colleges of physicians, associations of nursing, and board of practice for the various clinical specialties have the political authority to determine what must be known by the clinicians that they credential or license. The effect of the exercise of this political power is the creation of a set of PIO models that reflect, to a good first approximation, the common knowledge of clinicians who work together.

In fact, that is not quite right. Strictly speaking, at the level of field- or specialty-level governance, what gets standardized are *prototypes* of specific PIO models. These are the schemas or shells for PIO models that are useful in practice. This is an important distinction because neither patients nor medical institutions are standardized — and so another layer of governance over the expertise of clinicians comes from the culture of practice that has evolved and stabilized within the context of a specific medical institution. This informal — or anyway, less formal — source of political power “fills in” the schema that a clinician may have learned in school, or which reflects what the relevant credentialing authority expects clinicians to know, with details that constitute insights about, for instance, “how we can effectively treat CHF in this institution”^4^ or even “on *this* service” or “on *this* ward.” Suspect a STEMI?^5^ Then *this* is what we do — i.e. *this* PIO model is the one to rely upon when collecting information to confirm the initial hunch. Unclear whether the patient has CHF or CKF?^6^ Again: around here, here is how *we* sort this out — i.e. *these* are the PIO models that you can rely upon when collecting information in pursuit of a diagnostic model that is useful for the patient. Treating CRS while waiting for a diagnosis?^7^ Well, around here *this* is how we do it.

For example, an acute myocardial infarction results when one or more of the major coronary arteries becomes obstructed to the extent where the blood flow past the obstruction is inadequate to meet the demands of the heart. The standard treatment for a patient with an acute myocardial infarction is therefore removing the obstruction. This can be done with intravenous “clot busting” medication in the emergency department or via coronary angiography with stent placement in a cardiac catheterization lab. Whether a patient receives an immediate stent or not depends on whether the hospital they presented to has a cardiac catheterization lab and a 24 h on-call interventional cardiologist and team. Emergency medicine physicians working in remote areas of the country will provide patients with an acute myocardial infarction with intravenous medication and then immediately transfer them to the nearest available hospital that has a cardiac catheterization laboratory for stent placement. Whether the patient goes by ambulance or helicopter will depend on the patient’s condition, the weather, and the distance to the nearest accepting hospital. In contrast, a hospital in an urban area will almost always have a cardiac catheterization lab and on-call team to provide immediate coronary angiography and stent placement. Different institutions will therefore have different PIO models used to treat acute myocardial infarction, where the “ideal outcome” is that the obstruction is successfully removed.

We want to use the concept of federation of models, thus, to capture the important idea that any skilled clinician must know a fairly large number of these PIO models. But these models do not have semantic or syntactic properties that allow them to be aggregated or unified by application of some kind of mathematical or logical function, as the differing formats and contradictory content of these models prevents this. Strictly speaking, many of the models that make up a clinician’s expertise would be inconsistent with one another, if they could be formalized as sets of propositions. Because of this, their unity as a body of shared expertise really is more accurately seen as an important political achievement.

Here is another way of making the same point. Starting from the perspective of cognitive science, you may think that coordinated, efficacious clinical actions flow from one of three bases: shared knowledge of scripts or heuristics, shared knowledge of models, or shared knowledge of domain-general theories. Yet the first and the last option are not plausible. As illustrated by code blues, clinicians make coordinated counterfactual inferences fairly quickly about dynamic systems, implying that their thinking is guided by something more complex than a list or a flat tree structure. But a theory of cardiac resuscitation seems to be too complicated to be useful, even in non-emergency situations: such a theory would have to encode logical, semantic, or mathematical links between concepts for everything from telemetry readouts from ECGs to the microanatomy and physiology of the heart to differences in family dynamics. It would not just be a domain-general theory, technically, but an extremely unwieldy intra-domain theory. Unsurprisingly, it is hard to find any evidence of such a theory guiding the practice of clinicians.

But there is an even deeper reason to suspect that the first and the last kinds of explanations should be discounted: both overlook the importance of the reflexive relationship between *jurisdiction* and *configuration* in facilitating skilled clinical action. As we stressed above, both PIO models and diagnostic models are deeply situated. The reason we stressed this is that the material configuration of the clinical space is not accidental from the perspective of the clinician. As we mentioned above, to enact a code blue, as a matter of practical necessity you need the cardiac monitor, two large bore IV lines, IV fluids, ACLS drugs, advanced airway equipment, a defibrillator, and the ability to keep the patient’s body prone and firmly supported. But also, as a matter of practical necessity, you need the members of the code blue to have the same shared understanding of what they are doing: the cognitive systems of clinicians must be configured as well. They are as much a part of the material situation of the clinic as the stool used to assist with chest compression. The same is true about diagnostic models: as we have explained, the elements of a diagnostic model that is useful for generating beneficial interventions will encode any number of unique, situational elements — such as whether the patient has access to a fridge, the personality of the patient, and how likely it is that a patient will have durable access to a primary care physician.

That insight gets us to one of the deeper reasons why the concept of a federation of PIO models is useful. Political entities have jurisdictions: the space or spaces controlled by the political entity. And the reason we want to introduce the notion of a federation of PIO models — rather than just treat each useful PIO model as its own stand-alone epistemic object — is that all clinical spaces are configured for the enactment of a very large number of different PIO models, and, as discussed, all regulated forms of clinical expertise involve knowing the (roughly) same large number of PIO models. So, to capture this insight, it makes sense to talk about *federations* of PIO models that have jurisdiction over both the configuration of specific clinical spaces and the content of an individual’s clinical expertise. Medical institutions then host any number of different federations of PIO models, and give them the resources they need to shape the spaces and providers. A well-configured clinical space therefore makes the relevant PIO models feel concrete and intuitive to their clinical users. And because of this, a skilled clinician — or, more literally, a clinician who is useful in that space — must know a repertoire of PIO models, namely those which are in the federation with jurisdiction over where they practice.

Of course, this is not to deny that creativity — and other intellectual virtues that require autonomy for their exercise — play no role in providing expert clinic care. Creativity can find its application in the construction of diagnostic models, or the real-time reconciliation of failures of coordination that, if left to spin out on their own, may eventually generate an ethical failure or lapse in care, or in compensating for gaps in the relevant PIO models. But all the same, the point remains that clinicians are not free as individuals to construct their own bespoke PIO models: this would immediately destabilize the patterns of coordinated action that are critical to providing life-saving care. And even if a clinician did construct her own PIO model, there is no guarantee that it would be useful — or as useful as the alternatives: until it is federated with many other models, and the federation hosted by an institution that devolve to the federation jurisdiction over the configuration of material space, a model will be to such a clinician a deeply imperfect tool.

### 2.4 Inference to the best action

We have said enough now to be able to move to a presentation of how we believe inference to the best action should be conceptualized. To wit:

Inferences to the best action are insights into how to reconcile models with either old or new information that has emerged in the situation where goal-directed action is taking place. IBAs are actions that either maintain, restore, or improve coordination between the relevant models and reality.

IBAs establish — if only imperfectly — coordination, and *not* correspondence, between models and reality.

Thus: a diagnostic model of a patient’s CHF then leads to IBAs in the form of insight about how to treat the patient whose edema is not responding to first-line medications. A PIO model of a code blue leads to IBAs in the form of insights about how to respond to a faint pulse after several minutes of chest compressions. IBAs are judgments about how to ensure the flow of reality and the flow of clinical expertise remain more or less in alignment. For this reason, they are best thought of — and this is an important point — happening in sequences, rather than being one-off judgments. These chains of IBAs often aim to change the trajectory of ongoing (social, physiological, multi-level, multi-system) processes, causing at some point an inflection in the direction of the relevant processes: ROSC happens in a patient whose heart has stopped, or ventricular assist device restores cardiac output.

## 3 Can contemporary cognitive science explain clinical expertise

We have now shown how clinical expertise can be analyzed as the ability to generate inferences to the best action. It is time to switch to the critical part of our paper, in which we explore whether cognitive science can accommodate IBAs.

Our aim is to show that there is tension between both basically Humean and basically Bayesian theories of cognitive architecture, the accounts of expertise that are implied by these theories, and our account of clinical expertise as knowledge of diagnostic and PIO models that flows into inferences to the best action. To do this, we will first lay out a schematic representation of how Humean and Bayesian assumptions follow a common “structure” in generating an account of expertise. We will then assess how plausible this analysis of expertise is.

Here, then, is the relevant schema:

1. There are units of thought, and they are all of a single, common format.
2. Cognition is performing operations on the units of thought, the output of which is determined by either
  - (Humean) the unit’s location in a network containing only other units of thought and correlational relations defined over those units, or
  - (Bayesianism) involves attributing statistical properties to a new unit of thought according to calculations performed using statistical properties previously attributed to other units of thought, implemented often enough in correspondence with a Bayesian algorithm.
3. Causal knowledge is produced by either
  - (Humean) Sufficiently strong associative relations (i.e., constant correlations) between units of thought, or
  - (Bayesian) Inferring that a node represents an “intervention,” when the units of thought represent a Bayesian network (e.g., implying a definite joint probability distribution).
4. Turning expertise into skilled action consists in
  - Knowing a theory that corresponds to a domain, and which itself takes the form of a (potentially open-ended) set of whatever the units of thought are; and
  - Being able to generate new units of thought that are derived from applying either Humean or Bayesian operations to combinations of the relevant theory and new information; and where
  - That new information is integrated with the theory by a process of abstraction, in which it is formatted (or reformatted) so that it can be operated on by the Humean or Bayesian operations mentioned in 4b; and where
  - The output of 4.a-c. is a set of new units-of-thought that correspond to future state-of-affairs; and
  - Practical mean-ends reasoning uses these units-of-thought to identify and undertake actions that make these units-of-thought true, in the sense of correspondence truth.

We believe this list of assumptions approximately fairly well what a plurality of contemporary Humeans and Bayesians believe about cognition — see, for instance, (Fodor, 2000, 2003; Thagard, 2000, 2014; Griffiths et al., 2001; Tenenbaum et al., 2006, 2011; Gopnik, 2012; Gopnik and Wellman, 2012; Danks, 2014; Dammann et al., 2019). Of course, each assumption could be the subject of extensive debate. But our point here is not to try to refute or disprove any of these assumptions — again, our argument aims at a lower bar: we want to show that some of these assumptions are hard square with a realistic account of clinical expertise.

More explicitly, the critical argument is that both popular Humean and Bayesian theories of cognitive architecture yield implausible predictions about how clinical expertise informs and guides effective clinical action. As we interpret them in the schema above, both views represent expertise as first abstraction from a set of particular observations, then inference mediated by a domain-general theory and inferential operations that are either associationist (Humean) or Bayesian in their character, and finally application of the results of these inferences by way the construction of a set of propositions that are meant to correspond to states-of-affairs that have not yet been brought into existence but can through the exercise of means-end reasoning. But as we will try to show, when taken as an analysis of clinical expertise, it is implausible more or less on its own terms.

### 3.1 First critique

The first of these tensions arises from the fact that the standard account is that it predicts at least two inferences too many in the analysis of how clinical expertise is deployed.

According to the standard view, thus, in order to determine what to do, experts will have to engage in abstraction → inference → application sequence whenever an effective decision or action is required. Abstraction converts information that is already present into the whatever format units of thought have to take in order for them to be input into either Humean or Bayesian operations. Both Humeans and Bayesian require there to be some units-of-thought because each offers a “non-pluralistic” account of the cognitive mechanisms that implement cognition — ratiocinative thinking just is either the relevant Humean or Bayesian mechanisms in action.^8^ Inference is then what is detailed in 2a, 2b, 3a, and 3b, above. Application is then the “de-abstraction” of the results of inference, presumably by converting an intention into an action. Call this process an Ab → I → Ap sequence.

The critical point, then, is that to posit that Ab→ I → Ap sequences explain inferences to the best action seems to be positing at least two thoughts too many. What would normally be explained as a matter of perceptiveness and decisiveness in the case of a code blue that successfully resuscitates a patient, and what would normally be explained as a matter of curiosity and creativity in the case of diagnosing CHF in a patient with ambiguous signs and symptoms, are really both cases of the exercise of hidden ratiocination according to a logic that no one has ever been able to make explicit but is nevertheless a decisive and essential element in the delivery effective care.^9^ It certainly does not seem as if, when a code blue is called, clinicians figure out what to do minute-by-minute, and even second-by-second, by engaging in sequences of A →  I → A sequences. Positing these sequences seems unparsimonious. Likewise, it is hard to locate the domain-general theory that is used to develop a care plan that can be used to treat a particular patient’s CHF.

But there is a stronger argument for the same conclusion. The reason that we introduced the notion of a federation of PIO models is to call attention to how the physical configuration of a clinical space is designed to facilitate the application of PIO models. The models in some federation of PIO models and the configuration of the space where the models are useful are *tightly coupled* with one another: it really does matter that the step-stool be near the crash cart for the relevant PIO model of a code blue to be able to structure the model’s enactment. This tight coupling between physical configuration and the mental models of skilled clinicians, however, would be either unnecessary or epistemically irrelevant if, instead, we analyzed clinical expertise according to the standard view. We could just increase the content of the theory in order to account for either situational variability, or to otherwise give clinicians guidance for figuring out how to act across a comparatively much broader range of material situations. There would be no need for jurisdiction over both material configuration of clinical spaces and the epistemic configuration of clinicians themselves were it the case that clinical expertise were expression of suitably general theories.

Indeed, clinical spaces would be configured very differently if diagnostic decision making — let alone decision making in conditions of emergencies — if the cognitive processes underlying both have to proceed in a stepwise, “computational” fashion. The temporal dynamics of care are just too quick and the causal dynamics of care too complex for it to be plausible that clinicians use Ab →  I → Ap sequences. Clinician spaces would have to be dramatically simplified and the pace at which care is delivered dramatically slowed in order for there to be temporal compatibility between putative Ab →  I → Ap sequences and clinical reality.

As we noted above, the epistemic ideal for clinicians is to have knowledge of models that make any clinical problem situation feel concrete and manipulable; and if you have to stop and figure out what to do constantly, then uncertainty builds up with each educated guess or theoretical inference, and sooner or later that uncertainty crosses a threshold that renders you clinically ineffective. In fact, this insight is not ours: we owe it to an Emergency Medicine fellow in one of our classes, who was trying to explain to graduate students in biochemistry what becoming clinically competent involves. We add only to this that models can feel concrete only when situations are configured to allow them to feel that way — configuration that, again, would be redundant if clinical expertise usually took the form of knowledge of theories organized according to Humean or Bayesian principles.

### 3.2 Second critique

The second point of tension is easiest to grasp if we first explain why we stressed that clinical expertise flows from two different kinds of models. Models themselves need not be sets of propositions (Griesemer, 1991; de Chadarevian and Hopwood, 2004; Giere, 2010): sometimes they are causal graphs, sometimes they are networks, sometimes they are diagrams, and sometimes they are combinations of all of these, or combinations of both these and other kinds of information-carrying formats of representation. What’s more, scientific expertise sometimes consists in an understanding of a grab-bag of different models that are mathematically or logically inconsistent with one another, combined with knowledge of how to use these models to achieve any number of epistemic ends (Levins, 1966; Giere, 1985; Griesemer, 1996, 2004; Ankeny and Leonelli, 2011; Boyd, 2012; Leonelli, 2015).

Of course, our argument would be circular if we just asserted what, above, functions as a premise in our analysis of inference to the best action — namely that, to an important degree, clinical expertise is knowledge of different kinds of models. So, here, we will argue from two contextual observations to a similar conclusion: that clinical expertise is (substantially) knowledge of models, and not more or less unified theories that are expressed or represented in one common format, as per the standard view.

The contextual observations are these. First, nearly all clinicals will prefer new information that is useful over information that is consistent with previously formed beliefs. These judgments of usefulness are, we suggest, anticipations about how the information might be used to generate new inferences to the best action; indeed, to recognize that new information is clinically useful may be just the assessment that the information may be the basis for future IBAs once it is integrated into the clinician’s mental models. But we digress: the important point just here is that a preference for usefulness over consistency is hard to explain if clinical expertise takes the form of domain-general theories.

Next, the various formal conceptions of consistency that are used to define operationalizations of consistency — e.g. a union set formed out of two prior sets of well-formed formulas of a first ordered formal language that has at least one (non-empty) model (Pelletier, 1999; Prawitz, 2006) — play no role whatsoever in the production of nursing or medical scholarship, or in the training of clinicians. That is: there is no journal in either nursing or medicine (in contrast to philosophy) that is going to publish a paper that produces a “more consistent” or “no longer inconsistent” theory of congestive heart failure than some past theoretical formulation of the same, where some formal operationalization of consistency is the relevant analytical concept. Likewise, no one in any field of the health sciences can advance their career by making a model of a code blue explicit, translating it into a set of generic propositions, and then rendering that set of propositions consistent with another set of propositions that describe, e.g., how to apply the Glasgow Coma Scale. And it would be deeply weird, pedagogically speaking, to train either medical students or nursing students in formal conceptions of consistency, as this would be training that is irrelevant to all forms of practices.

Usefulness trumps consistency, and considerations of formal consistency are largely irrelevant to practice in nursing and medicine. But neither of these would be facts if expertise in these fields took the form of theories as per 3a. or 3b. above. Going in the other direction, however, neither observation is puzzling if, as per our analysis, clinical expertise consists in knowledge of models.

## 4 Conclusion

Can contemporary cognitive science explain clinical expertise? The answer might be “no,” because mainstream Humean and Bayesian views are committed to principles about the nature of cognition that yield implausible accounts of some paradigmatic cases of clinical expertise being put into practice. We have also provided the beginnings of an alternative account of clinical expertise, as knowledge of PIO and diagnostic models which are then reconciled with reality through the production of inferences to the best action. It is consequently interesting to ask what new theories of cognition might emerge if we begin by trying to understand clinical expertise starting from this perspective.

That said, we do want to acknowledge an important limitation to our argument. We have chosen two stylized examples that cannot be plausibly interpreted as skilled providers using ratiocination to derive implications of theories that then become both the causal and the justificatory basis of sequences of decisions that flow into effective actions. But it could be that similar efforts to analyze clinical expertise *in practice* contradict the assumption that our examples are stereotypical. Either way, we think that clinical expertise in a domain that deserves much closer study by mainstream cognitive science.

We would like to conclude by making a connection between the concepts of inference to the best action and inference to the best explanation. The latter concept, of course, refers to the ability to reliably identify true hypotheses on the basis of recognizing that some hypothesis offers — relative to some criteria — the best explanation of a set of observations compared to a set of other hypotheses. Philosophers and psychologists have suggested various criteria which determine either how the initial set of hypotheses is generated, or what makes one hypothesis in this set “the best” (Koslowski, 2012; Lipton, 2017; Lange, 2022). We suggest that one of the reasons an explanation may be selected as “the best” — considering the large number of philosophers who now believe that an important amount of scientific expertise consists in knowledge of models — is evidence that the explanation can be a source of productive chains of IBAs. Put another way around, the ability for a model to generate and support a productive chain of inferences to the best action may be a reason why scientists conclude that the model itself is the best explanation of whatever the model applies to.

We think therefore inference to the best action deserves further study, as it may play an important role in the cognitive science of expertise.

## Author contributions

MF wrote most of the first and second drafts, with NS and JDM contributing key sections. JDM and NS individually designed the several educational activities that allowed us test and refine the core ideas. All authors jointly conceptualized the core ideas in this manuscript.

## Footnotes

^1^Peirce writes: “abduction is the process of forming explanatory hypotheses. It is the only logical operation which introduces any new idea; for induction does nothing but determine a value, and deduction merely evolves the necessary consequences of a pure hypothesis” (Burks, 1946). These words are famously hard to interpret. But what we take it to mean is that a package of new ideas (whether or not they are technically a hypothesis) that offers a plausible — i.e. simple, or coherent, or compelling in light of other scientific considerations, which may actually be too long and too disperse to list, and where the “or” here is used inclusively — explanation of certain well-chosen examples has a degree of scientific confirmation. Other scientific theories should be reconciled with the abductively justified ideas.

^2^Indeed, we want to explicitly stress that what counts as a paradigm case of nursing expertise will be quite different from what counts as a paradigm case of medical expertise (Bender, 2018; Bender and Holmes, 2019; Fedyk, 2023).

^3^We want to stress that “multi-level” here means that these models incorporate dimensions or causes that can be found in all sorts of systems — or, more precisely, levels of partial but mostly stable for the foreseeable future organization. These models are messier than the (usually highly idealized) models of natural processes that frequent scientific textbooks. For more context, see Griesemer (1990, 1991) on models, and Fridland (2014, 2021), Fridland and Stichter (2021) on expert judgment and skill.

^4^Congestive heart failure.

^5^ST-elevation myocardial infarction, i.e., a heart attack.

^6^Chronic kidney failure.

^7^Cardiorenal syndrome.

^8^We ignore, for reasons of space, the possibility that the cognitive system is pluralistic in the sense of employing both Humean and Bayesian inferences in the core cognitive system.

^9^For discussion the different contextual factors which imply why it is implausible to speculate that both trainees and providers both are relying on some more or less explicit theory to navigate the code blue, please see (Yang and Howell, 2011; Huseman, 2012; Wehbe-Janek et al., 2012; Jackson and Grugan, 2015; Sachedina et al., 2019; Ka Ling et al., 2021).

## References

[ref1] Ankeny R. Chang H. Boumans M. Boon M. (2011). Introduction: philosophy of science in practice. Eur. J. Philos. Sci. 1, 303–307. doi: 10.1007/s13194-011-0036-4, PMID: 32168791

[ref2] Ankeny R. A. Leonelli S. (2011). What’s so special about model organisms? Stud. His. Phil. Sci. 42, 313–323. doi: 10.1016/j.shpsa.2010.11.039

[ref3] Bender M. (2018). Models versus theories as a primary carrier of nursing knowledge: a philosophical argument. Nur. Phil. 19. doi: 10.1111/nup.12198, PMID: 29057563

[ref4] Bender M. Holmes D. (2019). Reconciling nursing’s art and science dualism: toward a processual logic of nursing. Nurs. Inq. 26:e12293. doi: 10.1111/nin.12293, PMID: 30938042

[ref5] Boyd R. (2012). “Truth through thick and thin” in What is truth? ed. Schantz R. (Berlin, New York: de Gruyter).

[ref6] Burks A. W. (1946). Peirce’s theory of abduction. Philos. Sci. 13, 301–306. doi: 10.1086/286904

[ref7] Cartwright N. Psillos S. Chang H. (2001). “Theories of scientific method: Models for the physico-mathematical sciences,” in The Cambridge history of science volume 5: modern physical and mathematical sciences. ed. Nye M. J. (New York: Cambridge University Press).

[ref8] Dammann O. Poston T. Thagard P. (2019). “How do medical researchers make causal inferences?” What is scientific knowledge? An introduction to contemporary epistemology of science. 1st Edn. eds. McCain K. Kampourakis K. (New York: Routledge), 33–51. doi: 10.4324/9780203703809-3

[ref9] Danks D. (2014). Unifying the mind: cognitive representations as graphical models. New York, NY: MIT Press.

[ref10] de Chadarevian S. Hopwood N. (2004). Models: the third dimension of science. Redwood City, CA: Stanford University Press.

[ref11] Evans J. S. B. T. Stanovich K. E. (2013). Dual-process theories of higher cognition: advancing the debate. Perspect. Psychol. Sci. 8, 223–241. doi: 10.1177/1745691612460685, PMID: 26172965

[ref12] Fedyk M. (2023). Nursing science as the study of how to reconcile behavioral messiness with clinical norms and ideals. Stud. Hist. Phil. Sci. 99, 37–45. doi: 10.1016/j.shpsa.2022.12.006, PMID: 37001490

[ref13] Fodor J. A. (2000). The mind Doesn’t work that way: the scope and limits of computational psychology. New York, NY: MIT Press (Bradford book).

[ref14] Fodor J. A. (2003). Hume variations. New York, NY: Clarendon Press.

[ref15] Fridland E. (2014). They’ve lost control: reflections on skill. Synthese 191, 2729–2750. doi: 10.1007/s11229-014-0411-8

[ref16] Fridland E. (2021). Skill and strategic control. Synthese 199, 5937–5964. doi: 10.1007/s11229-021-03053-3

[ref17] Fridland E. Stichter M. (2021). It just feels right: an account of expert intuition. Synthese 199, 1327–1346.

[ref18] Giere R. N. (1985). Philosophy of science naturalized. Philos. Sci. 52, 331–356. doi: 10.1086/289255, PMID: 36070029

[ref19] Giere R. N. (2010). Scientific Perspectivism. Chicago, IL: University of Chicago Press.

[ref20] Gopnik A. (2012). Scientific thinking in young children: theoretical advances, empirical research, and policy implications. Science 337, 1623–1627. doi: 10.1126/science.1223416, PMID: 23019643

[ref21] Gopnik A. Wellman H. M. (2012). Reconstructing constructivism: causal models, Bayesian learning mechanisms, and the theory theory. Psychol. Bull. 138, 1085–1108. doi: 10.1037/a0028044, PMID: 22582739 PMC3422420

[ref22] Griesemer J. R. (1990). “Material models in biology,” in PSA: Proceedings of the biennial meeting of the philosophy of science association. Chicago IL: The University of Chicago Press. 1990, 79–93.

[ref23] Griesemer J. R. (1991). Must scientific diagrams be eliminable? The case of path analysis. Biol. Philos. 6, 155–180. doi: 10.1007/BF02426836

[ref24] Griesemer J. R. (1996). Periodization and models in historical biology. Mem. Calif. Acad. Sci. 20, 19–30.

[ref25] Griesemer J. R. (2004). “Three-dimensional models in philosophical perspective,” Models: The third dimension of science. eds. De Chadarevian S. Hopwood N. (Redwood City, CA: Stanford University Press), 433–442.

[ref26] Griffiths T. L. Kemp C. Tenenbaum J. B. (2001). “Bayesian models of cognition” in The Cambridge handbook of computational psychology. ed. Sun R. (New York, NY: Cambridge University Press), 59–100.

[ref27] Haraway D. (1988). Situated knowledges: the science question in feminism and the privilege of partial perspective. Feminist Stud. 14, 575–599. doi: 10.2307/3178066

[ref28] Harman G. H. (1965). The inference to the best explanation. Phil. Rev. 74, 88–95. doi: 10.2307/2183532

[ref29] Huseman K. F. (2012). Improving code blue response through the use of simulation. J. Nurses Staff Dev. 28, 120–124. doi: 10.1097/NND.0b013e3182551506, PMID: 22617782

[ref30] Jackson J. E. Grugan A. S. (2015). Code blue: do you know what to do? Nursing 45, 34–39. doi: 10.1097/01.NURSE.0000463651.10166.db 25807449

[ref31] Ka Ling F. Lim Binti Abdullah K. Seng Chiew G. Danaee M. Chan C. M. H. (2021). The impact of high Fidelity patient simulation on the level of knowledge and critical thinking skills in code blue management among undergraduate nursing students in Malaysia. SAGE Open 11:215824402110071. doi: 10.1177/21582440211007123

[ref32] Koslowski B. (2012). “Inference to the best explanation (IBE) and the causal and scientific reasoning of nonscientists,” in Psychology of Science: Implicit and Explicit Processes. eds. Robert W. P. Capaldi E. J. (Oxford University Press), 112–136.

[ref33] Lange M. (2022). Putting explanation back into “inference to the best explanation”. Nous 56, 84–109. doi: 10.1111/nous.12349

[ref34] Leonelli S. (2015). What counts as scientific data? A relational framework. Philos. Sci. 82, 810–821. doi: 10.1086/684083, PMID: 26869734 PMC4747116

[ref35] Levins R. (1966). The strategy of model building in population biology. Am. Sci. 54, 421–431.

[ref36] Lipton P. (2017). “Inference to the best explanation” in A companion to the philosophy of science. eds W. H. Newton-Smith (Oxford, UK: Blackwell Publishers Ltd), 184–193.

[ref37] Morewedge C. K. Kahneman D. (2010). Associative processes in intuitive judgment. Trends Cogn. Sci. 14, 435–440. doi: 10.1016/j.tics.2010.07.004, PMID: 20696611 PMC5378157

[ref38] Nersessian N. J. (2012). Engineering concepts: the interplay between concept formation and modeling practices in bioengineering sciences. Mind Cult. Act. 19, 222–239. doi: 10.1080/10749039.2012.688232

[ref39] Peirce C. S. (2021) in Logic of the future. ed. Pietarinen A.-V. (New York, NY: de Gruyter)

[ref40] Pelletier F. J. (1999). A brief history of natural deduction. Hist. Phil. Logic 20, 1–31. doi: 10.1080/014453499298165, PMID: 18050757

[ref41] Prawitz D. (2006). Natural deduction: a proof-theoretical study. Mineola, NY: Courier Dover Publications.

[ref42] Rogoff B. (1990). Apprenticeship in thinking: cognitive development in social context. New York, NY: Oxford University Press.

[ref43] Sachedina A. K. Blissett S. Remtulla A. Sridhar K. Morrison D. (2019). Preparing the next generation of code blue leaders through simulation: what’s missing? Simulation Healthcare 14, 77–81. doi: 10.1097/SIH.0000000000000343, PMID: 30395079

[ref44] Stanovich K. E. West R. F. (2000). Advancing the rationality debate. Behav. Brain Sci. 23, 701–717. doi: 10.1017/S0140525X00623439, PMID: 11301544

[ref45] Tenenbaum J. B. Griffiths T. L. Kemp C. (2006). Theory-based Bayesian models of inductive learning and reasoning. Trends Cogn. Sci. 10, 309–318. doi: 10.1016/j.tics.2006.05.009, PMID: 16797219

[ref46] Tenenbaum J. B. Kemp C. Griffiths T. L. Goodman N. D. (2011). How to grow a mind: statistics, structure, and abstraction. Science 331, 1279–1285. doi: 10.1126/science.1192788, PMID: 21393536

[ref47] Thagard P. (2000). How scientists explain disease. Princeton, NJ: Princeton University Press.

[ref48] Thagard P. (2014). The cognitive science of science: explanation, discovery, and conceptual change. Reprint Edn. New York, NY: MIT Press.

[ref49] Wehbe-Janek H. Lenzmeier C. R. Ogden P. E. Lambden M. P. Sanford P. Herrick J.. (2012). Nurses’ perceptions of simulation-based interprofessional training program for rapid response and code blue events. J. Nurs. Care Qual. 27, 43–50. doi: 10.1097/NCQ.0b013e3182303c95, PMID: 21849908

[ref50] Weisberg M. (2012). Simulation and similarity: using models to understand the world. New York, NY: Oxford University Press.

[ref51] Yang J. Howell M. D. (2011). Commentary: is the glass half empty? Code blue training in the modern era. Academic Med. 86, 680–683. doi: 10.1097/ACM.0b013e318217e969, PMID: 21613890

